# The surgery of intra-orbital hydatid cyst: a case report and literature review

**DOI:** 10.11604/pamj.2019.33.167.18277

**Published:** 2019-07-04

**Authors:** Kamal Chtira, Lamia Benantar, Houssaine Aitlhaj, Hasna Abdourafiq, Yassine Elallouchi, Khalid Aniba

**Affiliations:** 1 Neurosurgery Department, Ibn Tofail Hospital, University Hospital Mohammed VI, Marrakesh, Morocco; 2 Ophtalmology Department, Avicenne Miltary Hospital, University Hospital Mohammed VI, Marrakesh, Morocco

**Keywords:** Child, proptosis, hydatid cyst, orbit, surgery

## Abstract

Intra-orbital hydatid cyst is a very rare pathological entity that affects children and the young adults; it is secondary to the development in the orbit of the echinococcus granulosis tapeworm. Its frequency does not exceed 1% of all cases of hydatid disease. Clinical presentation of intra-orbital hydatid cyst is dominated by proptosis and a decrease in visual acuity, complete surgical excision is difficult, evolution is generally better when the treatment is early before the installation of irreversible optic atrophy. We report one case of a 3 years old girl operated for right intra-orbital hydatid cyst who presented with proptosis and blindness. Complete removal was difficult and puncture of the cyst was performed followed by excision of its membrane with good post-operative results. We also discuss the different epidemiological, clinical, radiological and therapeutic aspects of intra-orbital hydatid cyst and a review of literature of this rare pathology.

## Introduction

Intra-orbital hydatid cyst is rare, most commonly affecting children and young adults living in rural areas [1, 2]. It is secondary to development in the orbit of the *Echinococcus granulosus* tapeworm, whose definitive host are dogs and the intermediate host are sheep. In the parasitic cycle, man is only an accidental intermediate host. Morocco is an endemic country for hydatid cyst where hydatidosis is still prevalent and constitutes a non-exceptional cause of proptosis which represents the major clinical sign associated with a decrease in visual acuity or even monocular blindness and constitutes an emergency surgical treatment because, evolution without treatment will definitely alter the vision [3]. Through this observation we will recall the orbital localization of the hydatid cyst, which despite its rarity, is not exceptional, we will also discuss the different epidemiological, clinical, radiological and therapeutic aspects, especially the surgery of this cystic pathology.

## Patient and observation

**Figure 1:**
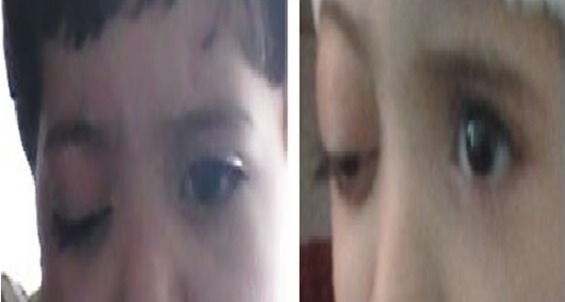
Preoperative image of the patient showing right proptosis and eyelid ptosis

**Figure 2:**
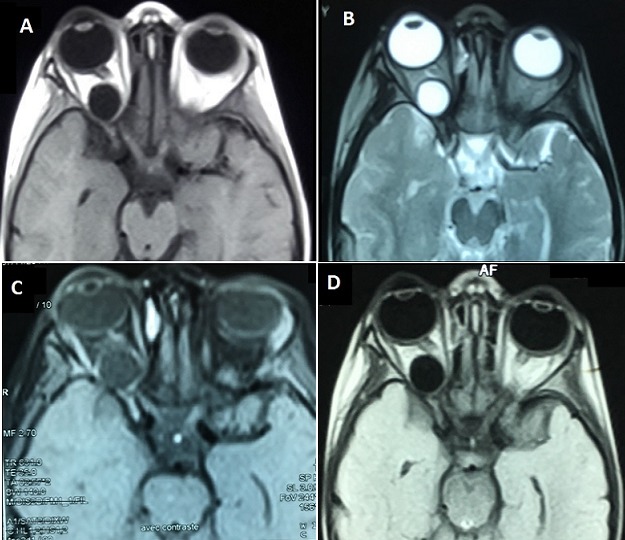
Brain MRI Axial sequences: T1 (A), T2 (B), T1 with contrast injection (C) and Flair Sequence (D) objectifying a rounded lesion that appears hypointense T1, hyperintense T2, well limited, thin walled Which takes slightly the contrast product (C), on the Flair sequence (D) it appears hypointense testifying to the liquid content of the lesion, which laterally compresses the optic nerve

**Figure 3:**
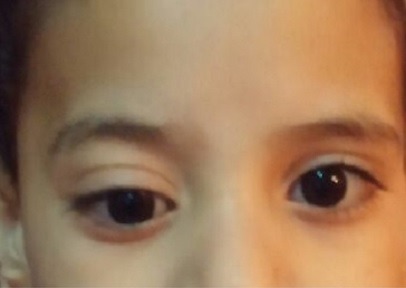
Image of the patient realized after 2 years of surgery showning ocular asymmetry and remarkable regression of proptosis and eyelid ptosis

**Figure 4:**
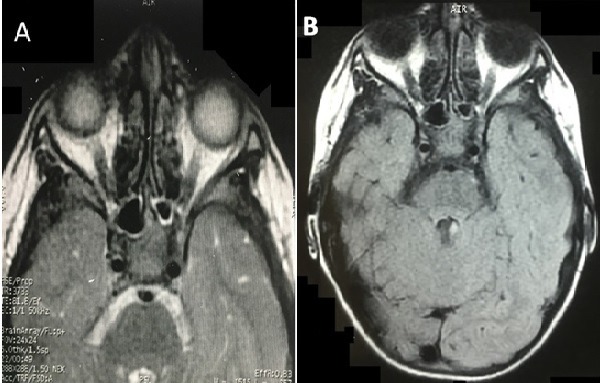
Postoperative brain and orbital MRI, axial T2 weighted sequence (A) and FLAIR (B) showing the total disappearance of the cyst with optic nerve decompression

A 3 years old girl born from non-consanguineous parents, without any neonatal suffering; with good psychomotor development, from a popular district in one of our cities, without any obvious contact with dogs; presented with exophthalmia associated with unilateral blindness evolving rapidly within 3 months. Clinical examination showed a non-pulsatile, painless, axial, irreducible exophthalmia with no sign of conjunctivitis or keratitis, and right monocular blindness, right ptosis; and fundal examination had objectified right papillary oedema (Figure 1). The rest of the clinical examination was normal. Brain and orbital magnetic resonance imaging (MRI) revealed an extra-connal right orbital lesion near the orbital apex, measuring 28 x 18mm, of oval shape, it appeared hypo-signal in T1 and hyper-signal in T2, limited by a thin wall which took contrast product, this lesion compressed the optic nerve towards the nasal region (Figure 2). Chest x-ray and abdominal ultrasound did not reveal any other localization. The patient was operated by performing a right extra-dural frontal approach, a cyst puncture was done in the first intention because the cyst was adherent to the neighbouring structures making its complete removal impossible, then a microscopic extirpation of the cystic membrane, combined with abundant washing by hypertonic serum to sterilize the cystic sit and reduce chances of dissemination. Histological examination was in favour of intra-orbital hydatidosis. Postoperatively, the patient was given an antihelminthic treatment of albendazole at a dose of 10mg per kilogram per day in 3 doses separated by 2 weeks; the evolution was marked by the significant regression of the exophthalmia and the gradual improvement of the visual acuity. The patient however still retains a right unilateral nasal hemianopia after two years of follow-up (Figure 3). An MRI done in the same period shows a cure of the patient by a complete disappearance of the cyst (Figure 4).

## Discussion

Intra-orbital localization of hydatid cyst is very rare; accounting for less than 0.8 to 1% of all hydatidosis localizations [4], our case is the first case of hydatid cyst operated in our university hospital. Usually, the orbital infestation is primitive, and is limited by an adventitia which becomes a thick shell very adherent to the surrounding tissues which makes dissection difficult. Intra-orbital hydatid cyst affects mostly children and the young adults between 2 and 20 years [4, 5]. It is always one-sided localization, predominant on the left and is often situated in the retro-bulbar, intra- or extra-conal, especially in the supero-medial angle, it is of variable volumes, however multi-cystic form is extremely rare [6, 7], in our case, we had unilateral intra orbital hydatid cyst localization which was very adherent to the neighbouring structures and total removal was difficult. The main clinical presentation is characterized by the appearance in all cases of the proptosis, which can be axial or not, painless, irreducible, non-pulsatile, non-blowing, and inflamed if cracking of the orbital hydatid cyst and is also associated with a decrease in the visual acuity [3-5]. The association with other localizations is very rare, but may be secondary to systemic dissemination [8], in our case the origin of the contamination was unknown and the proptosis and the blindness were the main clinical signs revealing the disease.

Radiological exploration is mainly based on magnetic resonance imaging (MRI) and computed tomography (CT), but also the ocular ultrasound can be requested, which can reveal a lesion in the form of a fluid mass , homogeneous with a thin wall, clear and regular limits, but sometimes the lesion can be heterogeneous if contains sediments or if infected [9]. With CT scan, the finding is a very evocative aspect: rounded or oval lesion, hypo-dense, homogeneous, with regular limits and its peripheral denser, (able to take contrast moderately). It makes it possible to specify the degree of proptosis, the topography of the intra- or extra-conical cyst and its repercussion on the elements of the eyeball and the orbit, as well as post-therapeutic surveillance [10]; however, MRI gives better analysis of the cyst with good spatial resolution, on T1 and T2 weighted sequences, the cyst appears hypo-intense on T1 and hyper-intense on T2, and wall enhancement after gadolinium injection [11, 12]. In our current practice the CT-Scan is requested before the MRI, concerning our case the patient is admitted after performing the brain MRI, the appearance found joins the characteristics described by literature. Several differential diagnoses can be evoked in the absence of epidemiological context and negative serology [2]; a reshuffled cavernoous angioma, a mucocele, a dermoid cyst, a colobomatous cyst, an epidermoid cyst and hematic cyst (post-trauma). Treatment of hydatid cyst is essentially surgical, avoiding the rupture of the cyst and its dissemination by making the easiest approach [4-13]. A three-month course of albendazole 15mg/kg is necessary when the cyst is removed with rupture in order to avoid dissemination of vesicles and prevent recurrence of the disease [14, 15]. In other cases, albendazole cures within one month and praziquantel of 2 weeks is instituted to sterilize the cyst, this decreases the risk of anaphylaxis, and reduces chances of post-operative recurrence. Use of hypertonic serum or 0.5% silver nitrate solution before opening the cystic cavity will tend to kill the female hydatids and prevent further spread or anaphylactic reaction, although the direct mortality due to echinococcosis is almost zero, morbidity can be very disabling [16]. In our experience we used hypertonic saline serum and oxygenated water to sterilize the cystic sit and we instituted medical treatment with albendazole at a dose of 10mg per kg per day for 28 days in 3 separate doses of 15 days and we had a long follow up to check for recurrence.

## Conclusion

Intra-orbital localization of hydatid cyst is very rare, clinical presentation is non-specific, radiological exploration is important and helps to make diagnosis; mainly MRI, and orbital CT scan, the treatment is exclusively surgical avoiding rupture of the cyst and it has a better prognosis with adequate management before the installation of visual field abnormalities and blindness. The diagnosis of orbital hydatid cyst should be considered in the differential diagnosis of unilateral proptosis, especially in patients who live in endemic countries.

## Competing interests

The authors declare no competing interests.
